# Supplementary material for: Quantifying the impact of public omics data
Source: Nat Commun. 2019 Aug 5;10:3512. doi: 10.1038/s41467-019-11461-w (PMC6683138; doi:10.1038/s41467-019-11461-w)
Supplement: Supplementary file 1 — Supplementary Information [file 41467_2019_11461_MOESM1_ESM.pdf]

Supplementary Information

Quantifying the impact of public omics data

Yasset Perez-Riverol [1, *], Andrey Zorin [1], Gaurhari Dass [1], Manh-Tu Vu [1], Pan Xu [2], Mihai Glont [1], Juan Antonio Vizcaíno [1], Andrew F. Jarnuczak [1], Robert Petryszak [1], Peipei Ping [3,4], Henning Hermjakob [1,2]

[1] European Molecular Biology Laboratory, EMBL-European Bioinformatics Institute (EMBL-EBI), Hinxton, Cambridge, UK.

[2] State Key Laboratory of Proteomics, Beijing Proteome Research Center, Beijing Institute of Lifeomics; National Center for Protein Sciences (The PHOENIX Center, Beijing), Beijing 102206, China

[3] Department of Physiology, Division of Cardiology, David Geffen School of Medicine at UCLA, University of California, Los Angeles, California, USA.

[4] Department of Medicine, Division of Cardiology, David Geffen School of Medicine at UCLA, University of California, Los Angeles, California, USA.

Table of Contents

Supplementary Note 1: Biological connection metric 3

Supplementary Note 2: Clustering based on metrics 6

Supplementary Note 3: Citing datasets 7

Supplementary Note 4: References between databases 8

Supplementary Note 5: Metrics Pipeline 9

Supplementary Note 6: Dataset replicate detection system 11

Supplementary Note 7: Claiming datasets system 12

Supplementary Note 8: Metrics per species 14

References 15

Supplementary Note 1: Biological connection metric

The *connections* metric is calculated by retrieving the references to the dataset from knowledgebases. For example, if a UniProt protein entry references a proteomics dataset, this counts as a connection to the dataset. The main idea is to keep track of all the biological knowledge generated by one omics dataset. This metric promotes the idea that the value of a dataset is related not only to the number of times it has been downloaded/reused/cited, but also how much biological knowledge (e.g. UniProt proteins, Ensembl genes, interactions or pathways) are supported by that dataset. **Table 1** shows all the knowledgebases data resources used by OmicsDI to compute the connections to omics datasets. The first column of the table includes the name of the data resource, the second column is the type of data and the last column is the number of entities it contains (by March 2019).

Table 1: Knowledge bases data resources included in OmicsDI.

Name of the Database	Type of Biological Knowledge	Number of Biological Entities
Assembly	Nucleotide sequences	144,952
Assembly scaffold (Release)	Nucleotide sequences	41,759,024
Baseline Expression Atlas Genes	Gene expression	775,676
ChEMBL Molecule	Bioactive molecules	1,735,442
ChEMBL Target	Bioactive molecules	11,538
Coding (Release)	Nucleotide sequences	356,332,997
DGVa	Genomes & metagenomes	42,992,846
Differential Expression Atlas Genes	Gene expression	376,636
Ensembl Gene	Genomes & metagenomes	3,113,803
Ensembl Genomes Gene	Genomes & metagenomes	175,370,460
Enzyme Portal	Enzymes	7,161
HGNC	Genomes & metagenomes	45,739
IMGT/HLA	Nucleotide sequences	13,641
IntAct Complexes	Molecular interactions	2,122
IntAct Interactions	Molecular interactions	528,593
IntAct Interactors	Molecular interactions	102,685
IntEnz	Enzymes	7,686
InterPro	Protein families	32,568
JPO	Protein sequences	2,795,178
KIPO	Protein sequences	518,927

Large assembly	Nucleotide sequences	44,032,859
Ligands	Bioactive molecules	19,672
Non-coding (Release)	Nucleotide sequences	19,045,545
PDBe	Macromolecular structures	107,958
PfamEntry	Protein families	16,712
PomBase	Genomes & metagenomes	6,995
RNAcentral	Nucleotide sequences	13,353,531
Reactome	Reactions & pathways	686,345
Rfam	Nucleotide sequences	2,309,950
Rhea	Reactions & pathways	43,456
Sequence (Release)	Nucleotide sequences	205,714,494
TreeFam	Protein families	15,736
USPTO	Protein sequences	4,077,368
UniProtKB	Protein sequences	108,184,003
WormBase ParaSite	Genomes & metagenomes	2,550,351

All these knowledge bases are indexed by the EBI Search resource (www.ebi.ac.uk/ebisearch/) [1]. The EBI search API is used to retrieve all the biological entities that reference the OmicsDI dataset or its corresponding publications (some databases use the publication identifier instead of the dataset accession). In addition, we add to the previous list the biological entities that have been reported by the dataset. By adding the list of referenced biological entities within the dataset we mitigate the time effect between the dataset publication and the use of the data in a knowledgebase.

For example, the dataset **E-MTAB-599** has 21,741 connections in EBI SEARCH (www.ebi.ac.uk/ebisearch/search.ebi?db=allebi&query=E-MTAB-599&requestFrom=searchBox); 115 Nucleotide sequences, 21,572 gene expression profiles, 54 Samples & ontologies mentions (lets called this **group A**). Then, we query the EBI Search for the manuscript associated with the dataset (https://www.ebi.ac.uk/ebisearch/crossrefsearch.ebi?db=europepmc&id=21921910&ref=genomes&requestFrom=relatedData-xref). The results showed 1,689,177 new connections from the Database from Genomics Variation Archive (**lets called this group B**). In addition, the 21,572 reported in the dataset considered (**lets called this group C**). The final connection metric number is the union of the three sets (**A** U **B** U **C**). By using the union of the three groups we avoid duplications of connections among providers (**Fig. 1**).

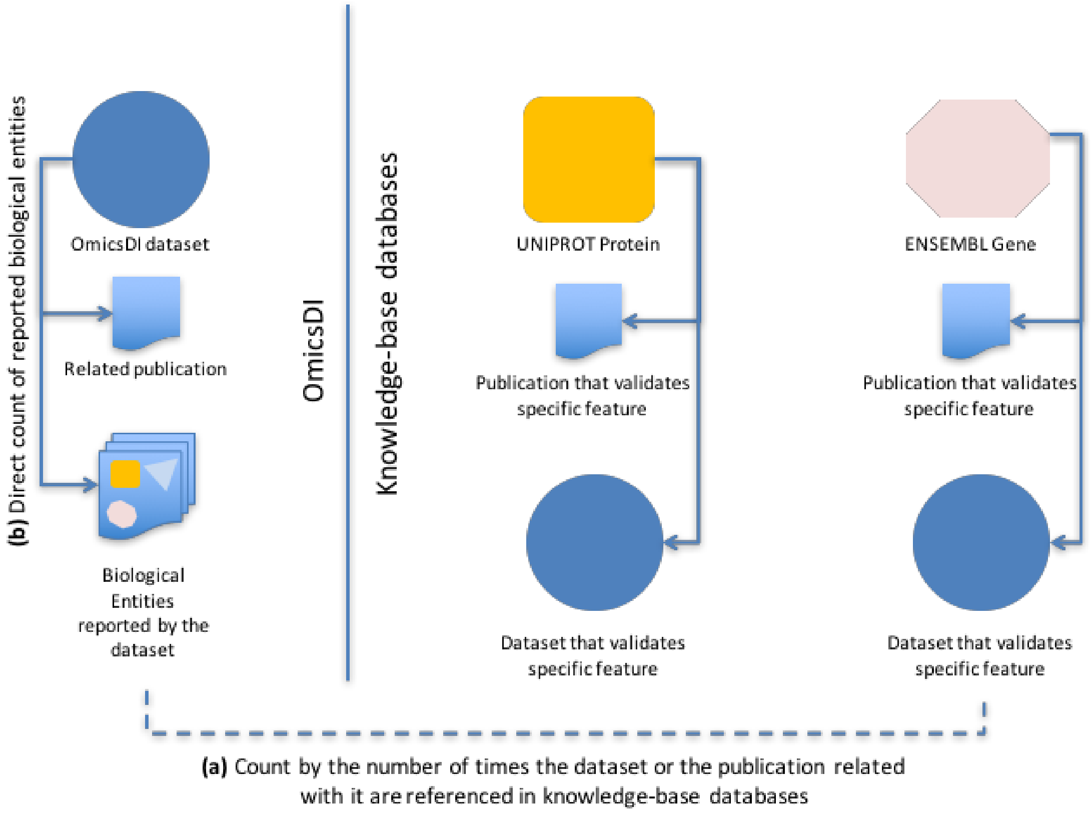

Figure 1: Schema of the reference system used in OmicsDI based on the EBI Search architecture. Each OmicsDI dataset is supported by a publication and a list of biological entities in knowledgebases (UniProt, Ensembl, etc). In the same way, entities in knowledgebases are supported by publications or datasets. (**a**) The connections pipeline in OmicsDI generates counts of connections to a dataset or its related publication, if referenced in a knowledgebase. In addition, (**b**) the pipeline uses the list of biological entities reported by the dataset to complement the previous lists in case the dataset hasn't been added to the knowledgebase. The final connection metric is the **union** of all the collected lists.

Supplementary Note 2: Clustering based on metrics

We have combined the data *citation* and *reanalyses* metrics using a simple *k-means* clustering algorithm to identify which are the datasets that are highly-cited, highly-reanalysed, moderately-cited, and moderately-reanalysed (**Fig. 2**). This simple classification enables users and services to search and find relevant datasets in a resource such as OmicsDI, with contains more than 100,000 datasets. The current results show that there are 113 (highly-cited), 682 (moderately-cited), 245 (moderately-reanalysed), 8 (highly-reanalysed) datasets.

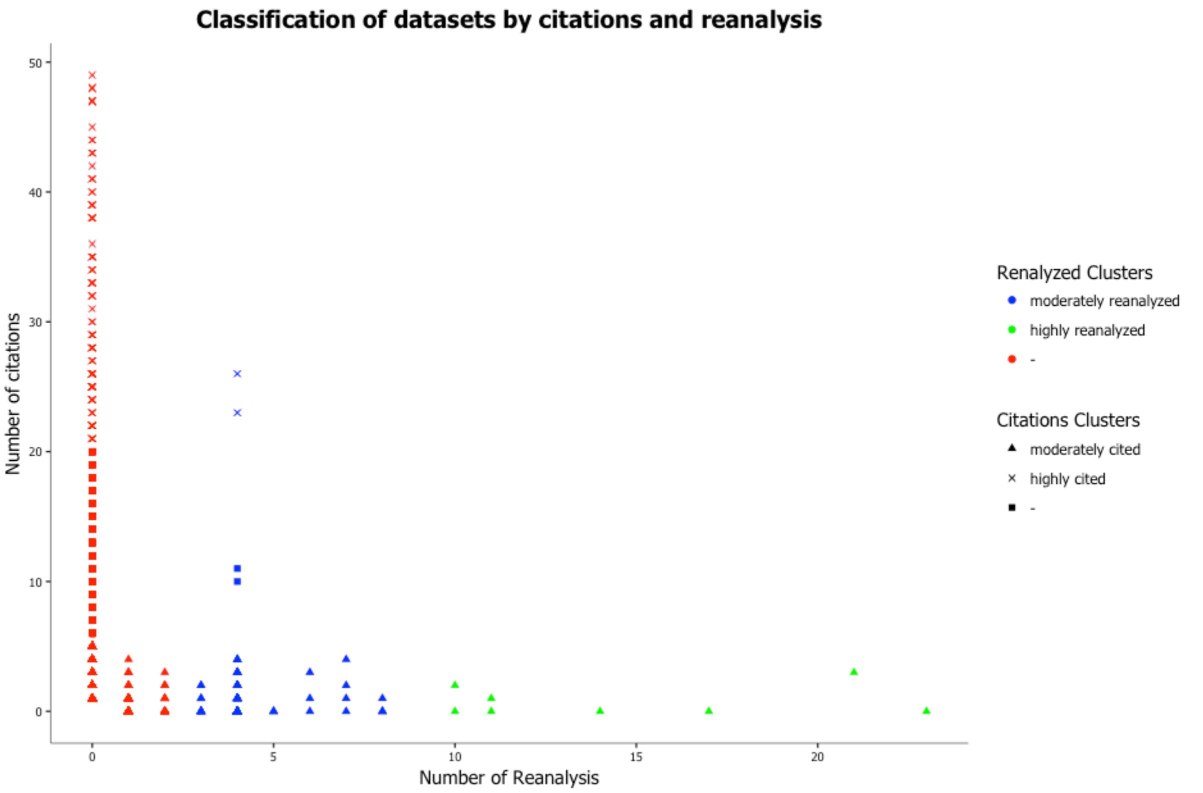

Figure 2: Classification of datasets into four different categories (highly-cited, highly-reanalysed, moderately-cited, moderately-reanalysed) using the *k-means* cluster algorithm.

Supplementary Note 3: Citing datasets

OmicsDI has implemented a simple visualization component (**Fig. 3**) that allows users to cite the corresponding dataset using the FORCE11 Data Citation Synthesis Group recommendations (http://www.dcc.ac.uk/resources/how-guides/cite-datasets)[3].

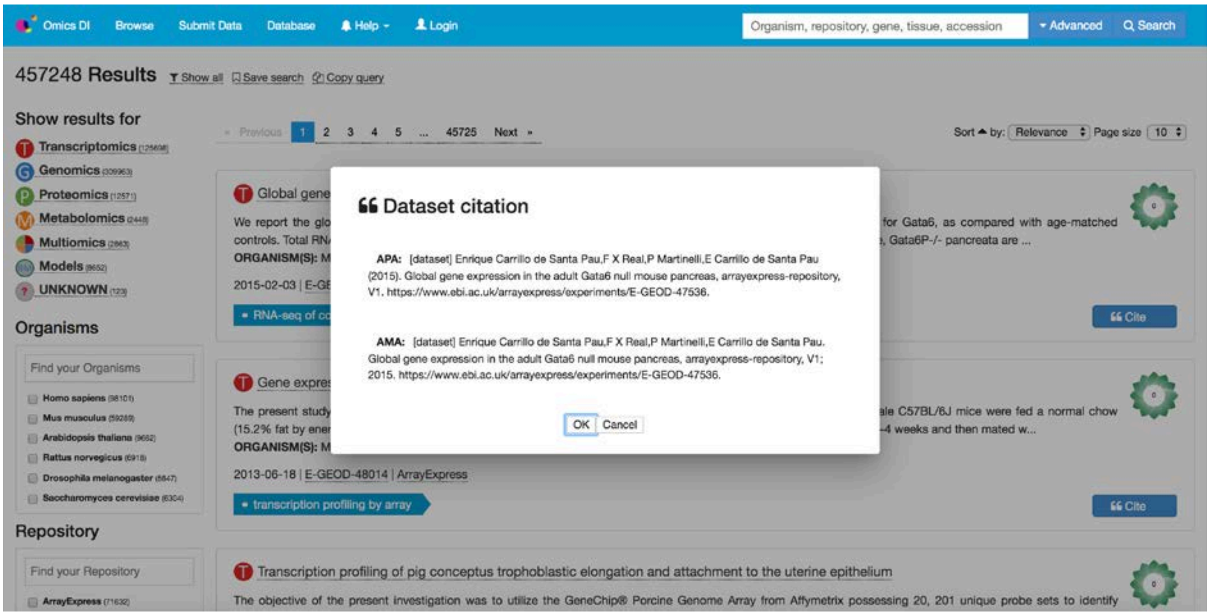

Figure 3: Dataset citations in OmicsDI can be exported in APA and AMA styles.

Supplementary Note 4: References between databases

Expression Atlas (www.ebi.ac.uk/gxa) adds to each reanalysed dataset the accession number and URL of the original ArrayExpress dataset. The reference to the original dataset in ArrayExpress enables OmicsDI to easily trace the number of reanalyses for each dataset.

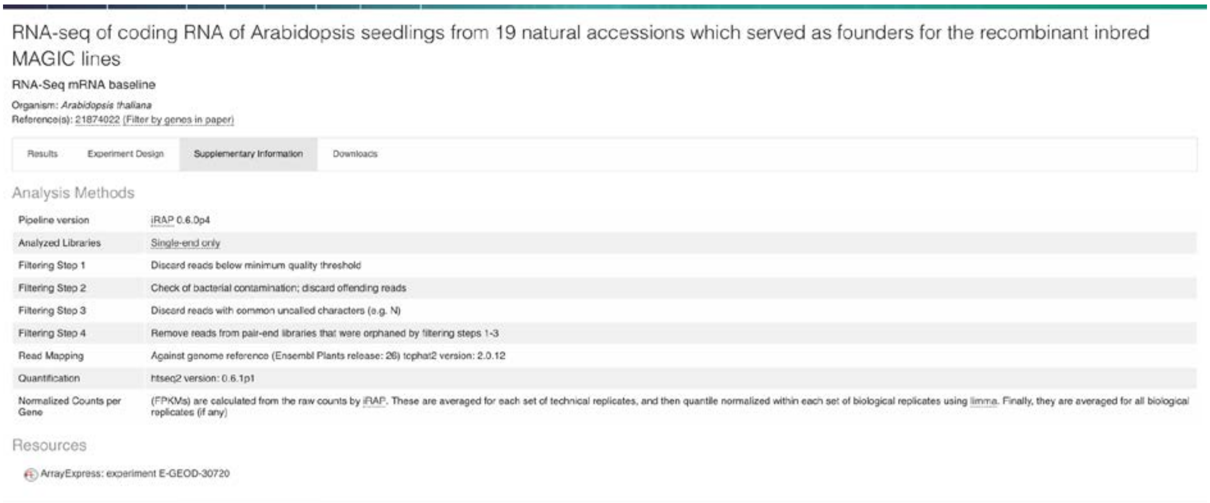

Figure 4: Expression Atlas' direct reference to the original re-used transcriptomics dataset originally deposited in ArrayExpress.

Supplementary Note 5: Metrics Pipeline

The metrics estimation pipeline is based on the Omics XML file format that is used to transfer datasets from each provider into OmicsDI (https://github.com/OmicsDI/specifications) (**Fig. 5**). **Fig. 5** shows how the data is imported from each provider into a central mongoDB database. An automatic pipeline is run to detect duplication and data replication across the resource between databases. For example, since ArrayExpress replicates all the datasets from GEO, the ArrayExpress dataset https://www.omicsdi.org/dataset/arrayexpress-repository/E-GEOD-46807 (accession number E-GEOD-46807) is a replicate of the GEO dataset GSE46807 and BioProject PRJNA202371. Some other types of replication are more complex to detect automatically and for this reason, a manual curation tool is also provided to merge datasets. Finally, the pipelines use the central mongoDB database, the EuropePMC API and the knowledgebases (e.g. Ensembl, UniProt, see Table 1) to compute/estimate the different metrics (**Fig. 9**).

The *reanalysis* count is computed using the reanalysis link provided on each OmicsDI XML file. If the dataset constitutes a *reanalysis* of a previous one, the XML file contains a reference to the original dataset. In the same way, each dataset contains the list of molecules reported in the analysis (e.g. UniProt protein, ChEBI or Ensembl gene identifiers) that are used to crosslink the dataset with the knowledgebases and compute the *connections* metric.

The *citations* metric is computed using the EuropePMC API (https://europepmc.org/RestfulWebService). Each dataset accession is searched using the API and the number of manuscripts that mentions the manuscript is retrieved (*citations* count). A precondition of this method is that the manuscript content needs to be accessible on EuropePMC and/or PubMed Central. The number of views is estimated by counting the number of times users access a dataset in the OmicsDI web interface but also the number of times the dataset is accessed using the API. By March 2019, for calculating the direct downloads of each dataset, major providers from EMBL-EBI including PRIDE, ArrayExpress, ENA, MetaboLigths and BioModels agreed to provide the number of downloads via the different file transfer methods: FTP, HTTP and Aspera. The number of downloads can be provided also using the OmicsDI XML file schema using the additional field *download_count*.

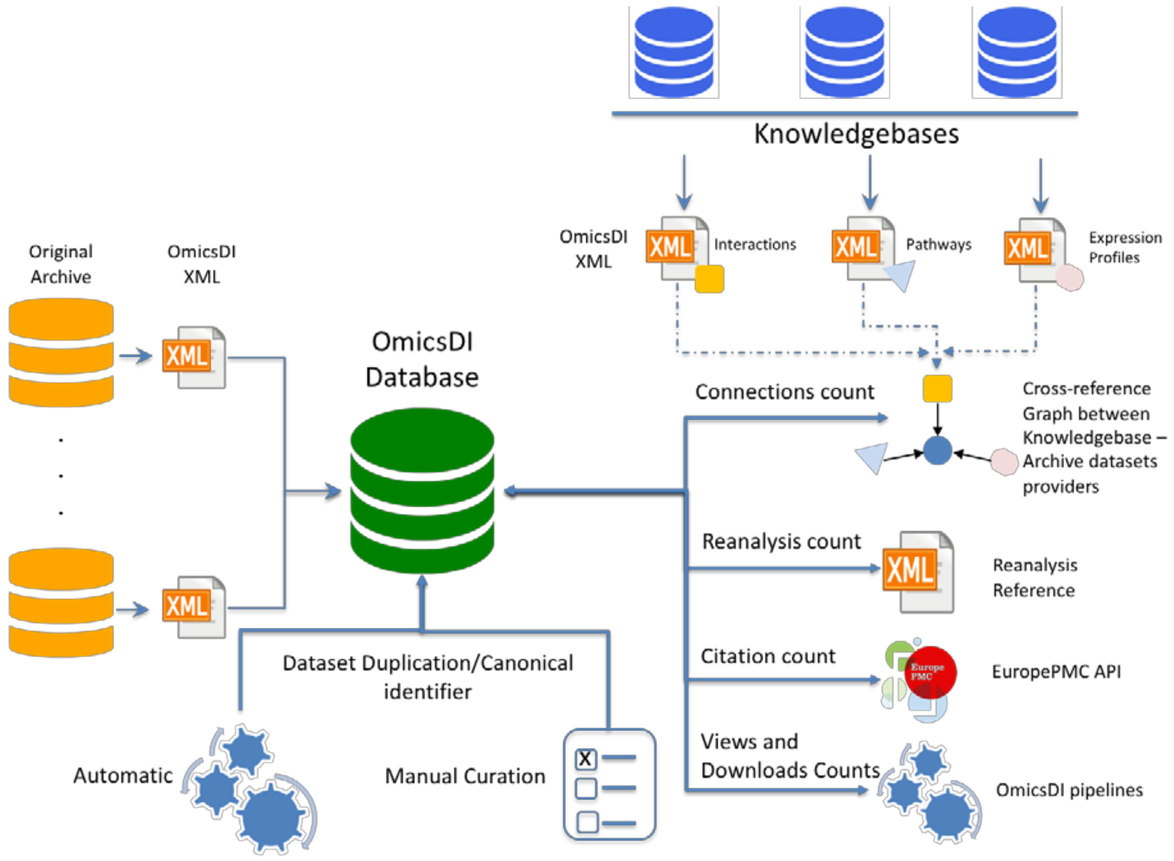

Figure 9: The metrics estimation pipeline is based on the Omics XML file format that is used to transfer datasets from each provider into OmicsDI. Data is imported from each provider into a central MongoDB database. An automatic pipeline is run to detect duplication and data replication across the resource. The pipelines use the central MongoDB database, the EuropePMC API and the knowledgebases (e.g. Ensembl, UniProt) to compute/estimate the different metrics.

Supplementary Note 6: Dataset replicate detection system

Dataset replication is considered a good practice because it guarantees that the dataset will be available even if one of the repositories needs to be closed down or runs out of funding. However, for indexing resources it is desirable to detect that the dataset is the same in the different providers to avoid redundancy. This is a complex task, the identifier of the dataset in the original provider is not always kept in the "replicate" versions. An automatic pipeline and a manual annotation system enable OmicsDI to remove duplicated datasets with potentially different identifiers (e.g. transcriptomics datasets available in ArrayExpress and Gene Expression Omnibus (GEO)). The pipeline designates one of the datasets as the canonical representation and annotates the rest of identifiers as additional secondary ones.

The following dataset (**Fig. 6**) was originally deposited in the Gene Expression Omnibus (GEO) (www.ncbi.nlm.nih.gov/geo/), replicated in the European Nucleotide Archive (ENA) (www.ebi.ac.uk/ena) and indexed by ArrayExpress (www.ebi.ac.uk/arrayexpress/) and BioProjects (www.ncbi.nlm.nih.gov/bioproject/). All those databases assign different identifiers to the same dataset, GSE59028, SRP044016, E-GEOD-59028 and PRJNA254155, respectively. The current OmicsDI pipeline automatically annotates all the identifiers for a dataset with replicates (ArrayExpress - E-GEOD-59028, **Fig. 6**). It also provides a web-interface to manually annotate those datasets that cannot be automatically "merged".

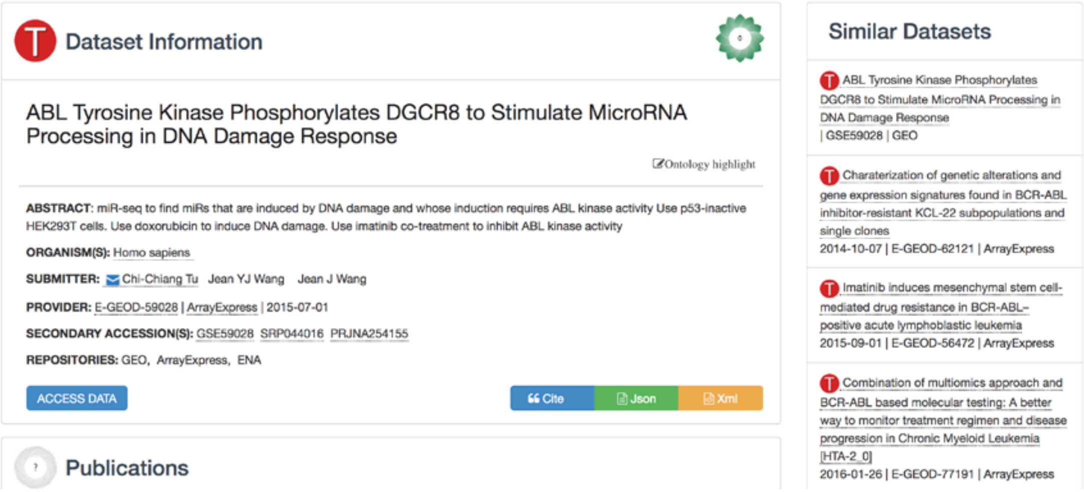

Figure 6: ArrayExpress dataset E-GEOD-59028. This dataset can be also found in the original database provider GEO (dataset identifier GSE59028), in the ENA database (identifier SRP044016) and in NCBI BioProjects (identifier PRJNA254155). The OmicsDI canonical identifier pipeline only represents one entry in the OmicsDI resource (in this case the ArrayExpress one) for the corresponding dataset but also provides all the alternative/additional identifiers in other data resources.

Supplementary Note 7: Claiming datasets system

OmicsDI has implemented a dataset claiming system that allows researchers to get credit for generating and sharing their datasets. The platform is built on top of three main components: i) a log-in system based on ORCID (**Fig. 7a**); ii) a dataset search-based claiming system that allows users to search and add the relevant datasets to their OmicsDI profiles (**Fig. 7b**); and iii) a user profile that enables users to update datasets in their OmicsDI profile (**Fig. 7a**) and synchronize them with ORCID (**Fig. 8b**). Additionally, as a key point, OmicsDI claimed datasets can be synchronized to the researcher's own ORCID profile, highlighting datasets there as a research product as well [2]. When the profile is synchronized with ORCID, the user's datasets are added to their ORCID profile. The claiming step is based on the *principle of trust* as in other popular analogous resources for publications such as Google Scholar or ResearchGate.

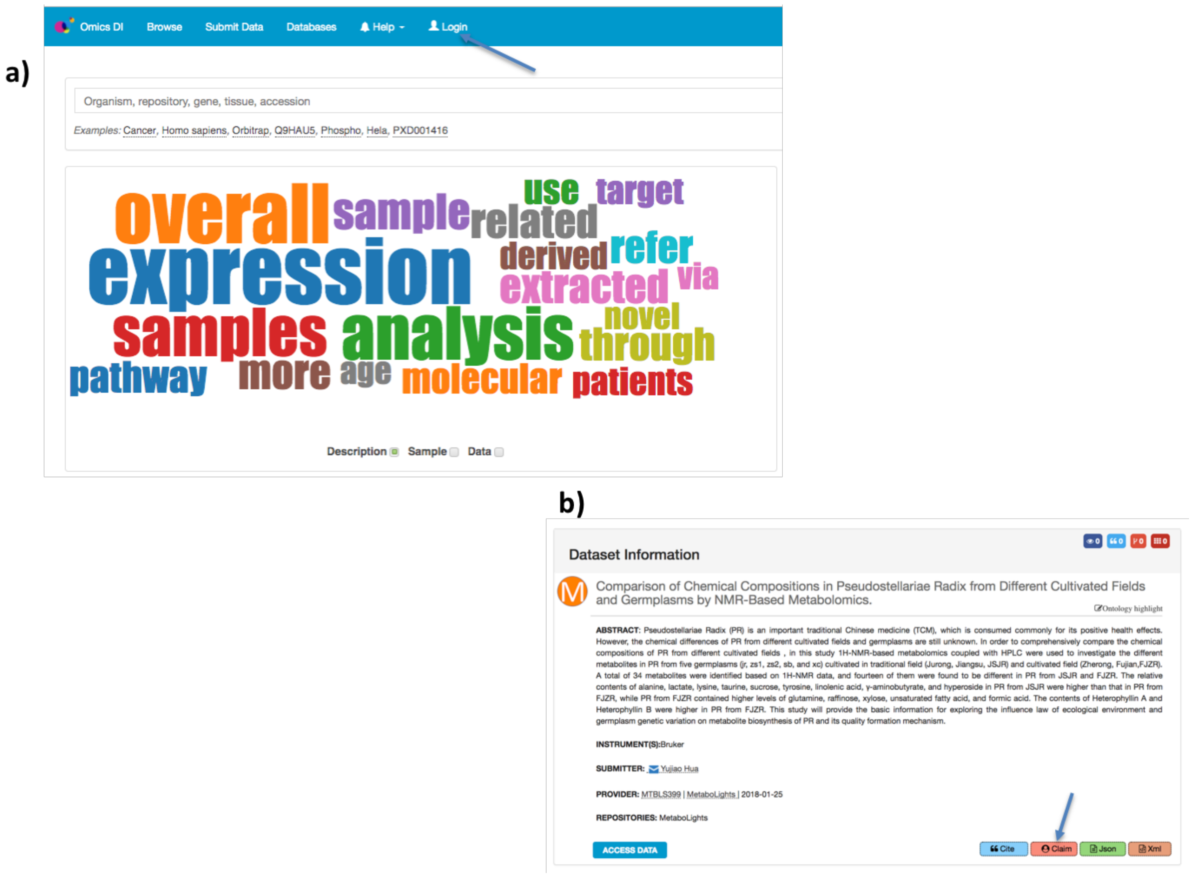

Figure 7: **(a)** OmicsDI provides a simple log-in and personal profile by using the ORCID authentication system. **(b)** When the researchers are logged in, a new option appears at the bottom of each dataset page, which allows them to claim the corresponding dataset.

a)

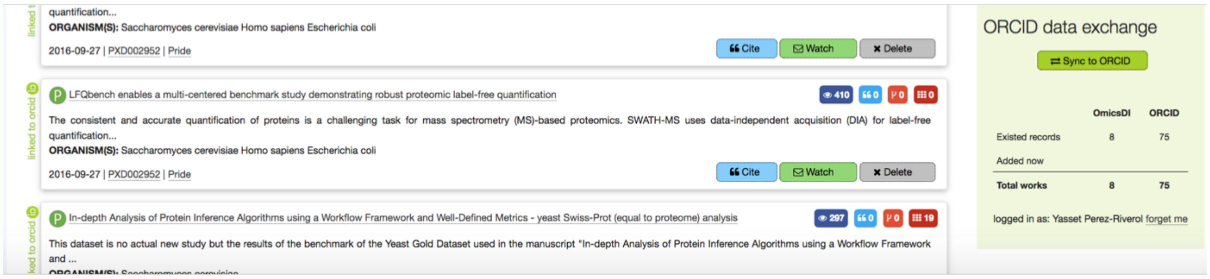

b)

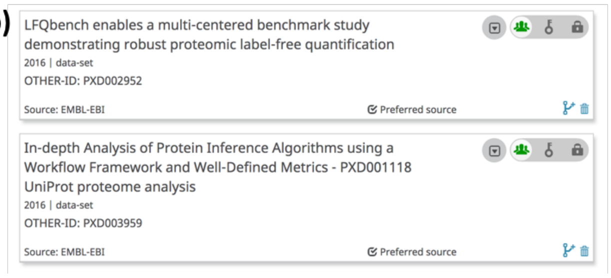

Figure 8: (**a**) Datasets claimed by a specific user are listed in their OmicsDI profile. OmicsDI enables users to update their profile. A profile can be made public and the URL can be shared in the public domain to demonstrate the impact of the author's datasets. **(b)** Each researcher's dataset list can be synchronized with their own ORCID profile.

Supplementary Note 8: Metrics per species.

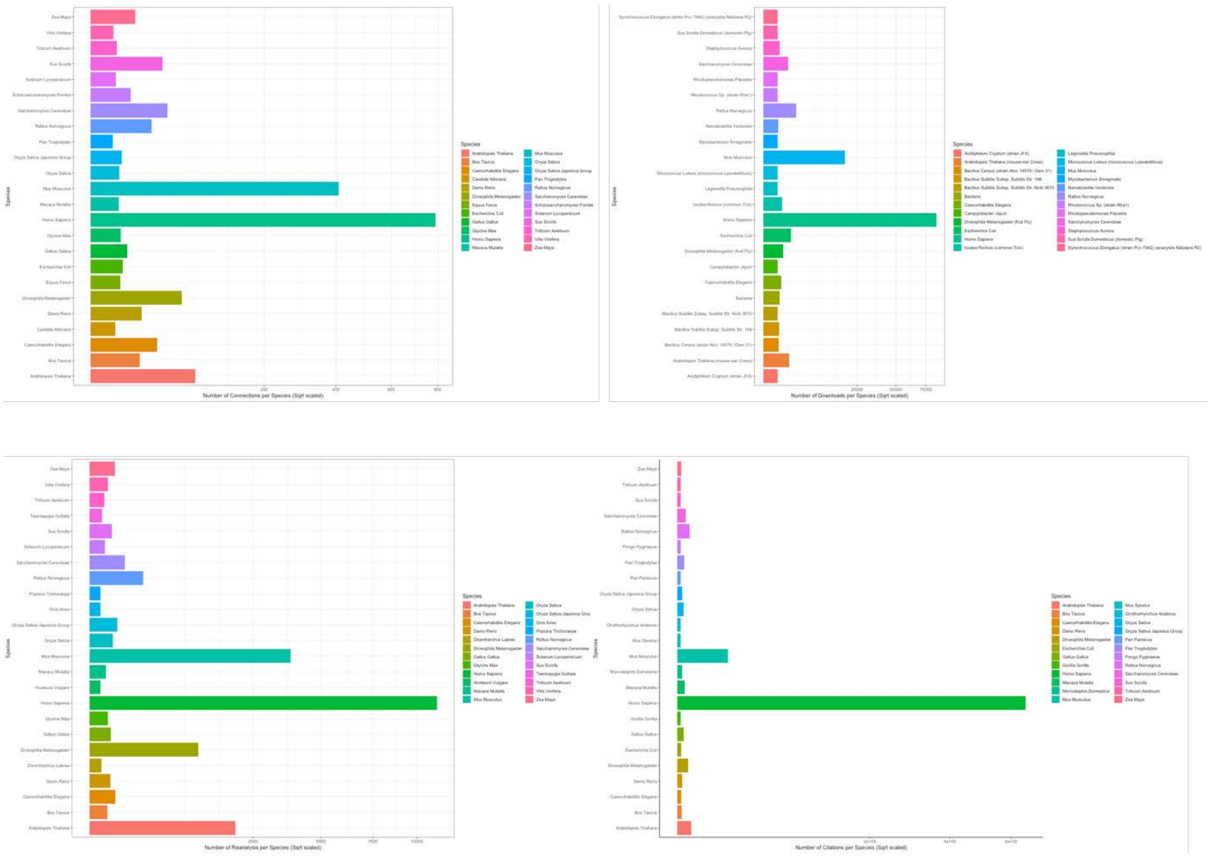

Figure 9: Distribution of metrics (*connections, downloads, reanalyses* and *citations*) per species.

References

1. Park, Y.M., Squizzato, S., Buso, N., Gur, T. & Lopez, R. The EBI search engine: EBI search as a service-making biological data accessible for all. *Nucleic Acids Res* **45**, W545-W549 (2017).
2. Haak, L.L., Fenner, M., Paglione, L., Pentz, E. & Ratner, H. ORCID: a system to uniquely identify researchers. *Learned Publishing* **25**, 259-264 (2012).
3. Ball, A. & Duke, M. How to cite datasets and link to publications. (Digital Curation Centre, 2011).
